# Pharmacist-Driven Culture and Sexually Transmitted Infection Testing Follow-Up Program in the Emergency Department

**DOI:** 10.3390/pharmacy8020072

**Published:** 2020-04-23

**Authors:** Stephanie C. Shealy, Christine Alexander, Tina Grof Hardison, Joseph Magagnoli, Julie Ann Justo, Caroline Derrick, Joseph Kohn, Hana Rac Winders, Troy Privette, Majdi N. Al-Hasan, P. Brandon Bookstaver

**Affiliations:** 1 Department of Pharmacy, Prisma Health Richland Hospital, Columbia, SC 29203, USA; shealysc@email.sc.edu (S.C.S.); christine.alexander@prismahealth.org (C.A.); tina.hardison@prismahealth.org (T.G.H.); justoj@cop.sc.edu (J.A.J.); joseph.kohn@prismahealth.org (J.K.); 2 Department of Clinical Pharmacy and Outcomes Sciences, University of South Carolina College of Pharmacy, Columbia, SC 29208, USA; magagnol@mailbox.sc.edu (J.M.); hwinders@cop.sc.edu (H.R.W.); 3 Department of Medicine, University of South Carolina School of Medicine, Columbia, SC 29209, USA; caroline.derrick@uscmed.sc.edu (C.D.); majdi.alhasan@uscmed.sc.edu (M.N.A.-H.); 4 Emergency Department, Prisma Health Richland Hospital, Columbia, SC 29203, USA; priv@aol.com

**Keywords:** pharmacy resident, rapid diagnostic technology, urinary tract infections, antimicrobial stewardship

## Abstract

Expanding pharmacist-driven antimicrobial stewardship efforts in the emergency department (ED) can improve antibiotic management for both admitted and discharged patients. We piloted a pharmacist-driven culture and rapid diagnostic technology (RDT) follow-up program in patients discharged from the ED. This was a single-center, pre- and post-implementation, cohort study examining the impact of a pharmacist-driven culture/RDT follow-up program in the ED. Adult patients discharged from the ED with subsequent positive cultures and/or RDT during the pre- (21 August 2018–18 November 2018) and post-implementation (19 November 2018–15 February 2019) periods were screened for inclusion. The primary endpoints were time from ED discharge to culture/RDT review and completion of follow-up. Secondary endpoints included antimicrobial agent prescribed during outpatient follow-up, repeat ED encounters within 30 days, and hospital admissions within 30 days. Baseline characteristics were analyzed using descriptive statistics. Time-to-event data were analyzed using the Wilcoxon signed-rank test. One-hundred-and-twenty-seven patients were included, 64 in the pre-implementation group and 63 in the post-implementation group. There was a 36.3% reduction in the meantime to culture/RDT data review in the post-implementation group (75.2 h vs. 47.9 h, p < 0.001). There was a significant reduction in fluoroquinolone prescribing in the post-implementation group (18.1% vs. 5.4%, p = 0.036). The proportion of patients who had a repeat ED encounter or hospital admission within 30 days was not significantly different between the pre- and post-implementation groups (15.6 vs. 19.1%, p = 0.78 and 9.4% vs. 7.9%, p = 1.0, respectively). Introduction of a pharmacist culture and RDT follow-up program in the ED reduced time to data review, time to outpatient intervention and outpatient follow-up of fluoroquinolone prescribing.

## 1. Introduction

In 2017, the Joint Commission established the requirement for hospitals and nursing homes to have an antimicrobial stewardship program (ASP) [1]. Although ASPs have been primarily focused in the inpatient setting, evidence demonstrates that these programs can reduce unnecessary antimicrobial prescriptions and improve timely antimicrobial selection in the outpatient setting, including in the emergency department (ED) [2,3]. Optimized antimicrobial use has been widely associated with improved clinical outcomes and reduced adverse drug events (ADEs), including *Clostridioides difficile* infection [4,5,6].

Incorporation of a stewardship pharmacist into culture and sexually transmitted infection (STI) testing follow-up may be an effective method for ASP and pharmacy service expansion to the outpatient setting [7,8,9,10,11,12,13]. In addition to improved antimicrobial selection, culture follow-up programs that involve a pharmacist have been shown to reduce subsequent ED visits and reduce the time to result review and patient notification when compared to programs without a pharmacist [7,8,9,13]. The introduction of rapid diagnostic technology (RDT) for routine bacterial cultures and STIs has provided another pathway for early intervention in discharged patients [14,15]. Although guidelines recommend empiric treatment for suspected STIs, patients may require outpatient follow-up if the empiric treatment was not appropriate based on the STI result or if the patient was not empirically treated due to low suspicion [16]. Pharmacist involvement in outpatient follow-up specifically for untreated STIs is not well reported.

Integration of ASP activities into routine ED practices may be challenging in settings of high patient volume and limited personnel, especially for discharged patients where challenges in follow-up occur. Utilizing pharmacy trainees (e.g., residents) or non-ED personnel may be required in some institutions. Currently, our institutional model for follow-up of discharged ED patients with positive cultures is driven primarily by administrative support staff and cross-cover physicians. Potential areas for optimization were identified including delay in time to review and desire for stewardship expertise in the process using an interdisciplinary approach. Thus, a pilot pharmacist-driven culture and RDT follow-up program was implemented in November 2018.

## 2. Materials and Methods

### 2.1. Study Setting

This study took place at a tertiary teaching hospital ED, which had approximately 95,000 patient visits in 2018. At this institution, ED pharmacists are involved in antimicrobial decision support regarding cultures only when prompted by physicians prior to patient discharge. The current culture follow-up practice for patients discharged from the ED involves communication among ED administrative support staff (two individuals without formal clinical training) and physicians. The administrative support staff are notified of all positive cultures and STI results for patients discharged from the ED. All positive results that return during the previous 24 h are reviewed by the administrative staff and acted upon the following morning. This staff review identifies discrepancies that may require outpatient follow-up and brings these specific cases to the attention of the covering ED physician. For cultures, the staff primarily interpret upon finalization of the culture and identify discrepancies, including a ‘bug–drug’ mismatch with the prescribed antimicrobial and the susceptibility report or no antimicrobial prescribed for the positive culture. Positive STI RDT which was not empirically treated in the ED is also identified by the staff and brought to the physician for review. The covering physician, who also has acute patient responsibilities in the ED, is tasked to review the cases selected by the staff and communicate the intervention plans to the staff each morning. The staff then contact the patient to carry out the outpatient follow-up. If the patient is not able to be contacted via telephone, a final attempt to contact the patient is made through mailing of a letter.

Through implementation of a pharmacist-driven workflow, a PGY-1 pharmacy resident with antimicrobial stewardship training implemented a live-alert system through an integrated clinical decision support software (TheraDoc^®^) for all culture and STI RDT updates and reviewed these results daily for patients who were discharged from the ED. A background and training with the antimicrobial stewardship and support team provided the confidence and expertise for the pharmacy resident to review and triage results through the alerts, identify discrepancies that may require outpatient follow-up, comprise a recommendation with the ED pharmacist, and communicate and implement a plan in collaboration with the ED physician. The recommendations were based on local antimicrobial prescribing guidelines, produced by the local antimicrobial stewardship and support team, and national clinical management guidelines, when available. The pharmacy resident executed the follow-up plan that was agreed upon by the physician, ED pharmacist, and pharmacy resident. A representation of the pharmacists’ workflow during the pilot culture follow-up program is located in Appendix A. The pilot, pharmacist-driven program was executed from 19 November 2018 through 15 February 2019.

### 2.2. Study Design

This was an IRB-approved, single-centered, pre- and post-implementation cohort study. Cultures drawn during the pilot period were classified as the post-implementation group. The comparator group, i.e., the observational pre-implementation group, included cultures drawn during the 90 days prior to implementation (21 August 2018 through 18 November 2018). All positive urine, blood, wound, and stool cultures and positive STI RDT for *Neisseria gonorrhoeae*, *Chlamydia trachomatis*, and *Trichomonas vaginalis* which originated in the ED were screened for inclusion. Patients aged ≥18 years who were discharged from the ED with positive cultures and/or positive STI RDT and for which an outpatient follow-up was attempted were included in the study. Patients who expired in the ED, were admitted to the hospital, or transferred to another institution were excluded.

The primary endpoints were time from ED discharge to result review and time from ED discharge to completion of outpatient follow-up. Secondary endpoints included time from ED discharge to time to first patient contact attempt, percentage of patients who had a repeat ED encounter within 30 days of the index encounter, percentage of patients who were admitted within 30 days of the index encounter, and antimicrobial prescribed during outpatient follow-up.

Descriptive statistics were used to characterize patients and culture types. The primary endpoints of time to result review and time to outpatient intervention completion were analyzed using the Wilcoxon signed-rank test. Chi-square or Fisher’s exact tests were used to examine the differences in secondary outcomes in the pre- and post-intervention groups. An a priori sample size calculation concluded that 157 patients were required to provide 95% power to detect a 20% difference in the primary endpoint of time to review of positive culture or STI RDT.

## 3. Results

**Figure 1:**
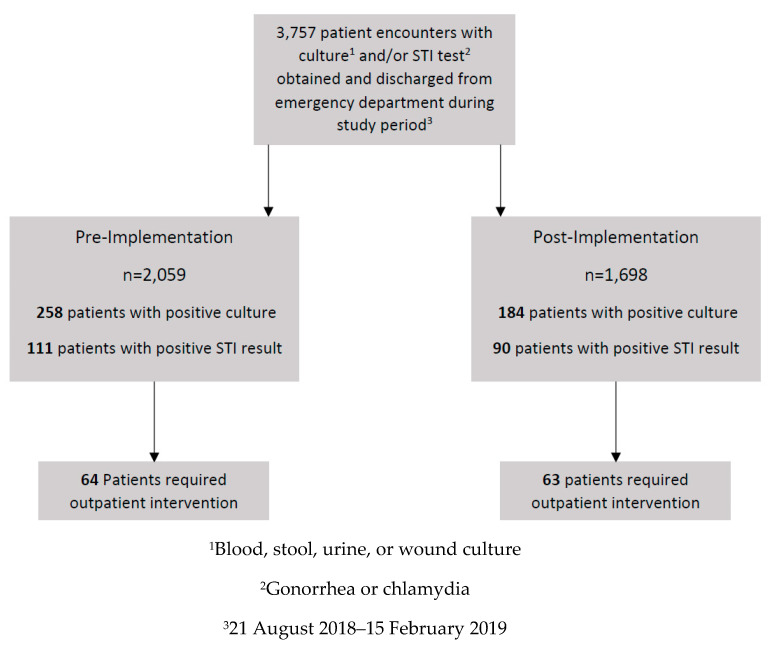
Screening and Enrollment.

Of the 3757 patients discharged from the ED with cultures obtained, 127 patients were included—64 patients in the pre-implementation group and 63 patients in the post-implementation group (Figure 1). The primary reason for exclusion (n = 337) was that outpatient follow-up was not required as initial ED treatment plan was appropriate based on culture and/or STI RDT result (i.e., patient empirically treated for STI in the ED, pathogen on culture susceptible to discharge antimicrobial and discharge antimicrobial dosing and duration sufficient, etc.). Baseline characteristics are summarized in Table 1. The most common result type was urine cultures (63.6%), followed by STI RDT (30.2%). Most patients were not prescribed an antimicrobial upon ED discharge (pre, 59.4% vs. post, 60.3%).

**Figure 2:**
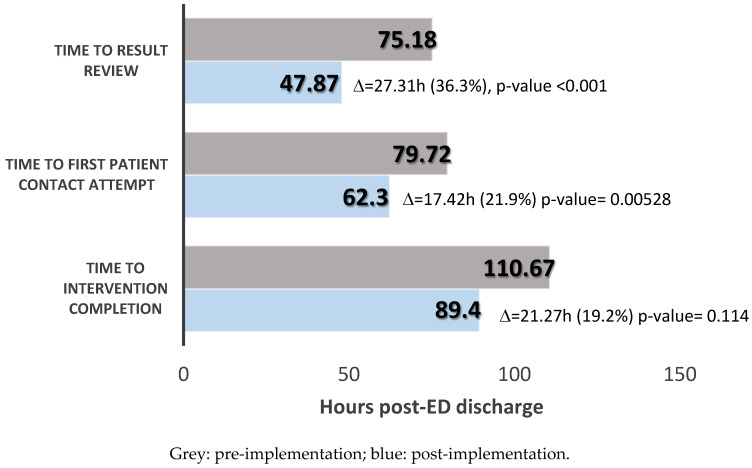
Time-to-event data.

Time-to-event data are summarized in Figure 2. The mean time to result review and mean time to first attempt at patient contact were significantly reduced in the pharmacist-driven program by 27.3 h and 17.4 h, respectively (75.2 h vs. 47.9 h, p < 0.001; 79.7 h vs. 62.3 h, p = 0.005). The mean time to completion of outpatient intervention decreased, although this was not statistically significant (110.7 h vs. 89.4 h, p = 0.114).

**Figure 3:**
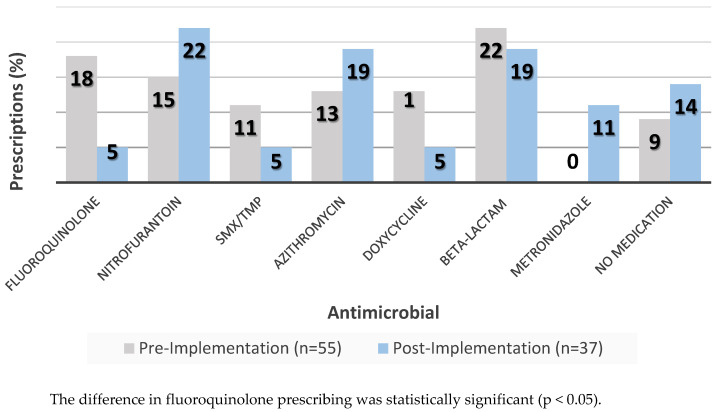
Medications Prescribed During Outpatient Follow-Up.

As demonstrated in Table 2, the most frequent intervention for both groups was initiation of active therapy (39.1% vs. 30.2%, p = 0.39), followed by modification of previously prescribed therapy (37.5% vs. 22.2%, p = 0.09). The post-implementation group had a significantly higher rate of sending a letter as a final outpatient intervention attempt than the pre-implementation group (4.7% vs. 19.1%, p = 0.026). Among patients who had initiation or modification of previously prescribed therapy, there was a significant reduction in proportion of patients prescribed a fluoroquinolone in the post-implementation group (15.6% vs. 3.2%, p = 0.036). Rates of prescribing for other antimicrobials and classes were similar, as demonstrated in Figure 3.

The proportion of patients who had a repeat ED encounter within 30 days was 10/64 (15.6%) in the pre-implementation group and 12/63 (19.1%) in the post-implementation group (p = 0.78) (Table 3). Of the 10 patients who experienced a repeat ED encounter in the pre-implementation group, five cases were related to the index ED encounter, with no related cases in the post-implementation group. The proportion of patients who experienced a hospital admission within 30 days in the pre-implementation group was 6/64 (9.4%) and in the post-implementation group was 5/63 (7.9%) (p = 1.0). Of the six patients in the pre-implementation group who experienced a hospital admission, two admissions were related to infection, and of the five patients who experienced a hospital admission in the post-implementation group, one admission was related to infection. For the pre-implementation group, one patient was admitted intentionally as a result of the outpatient intervention and three patients were admitted for this reason in the post-implementation group. The reason for intentional admission for these patients was positive blood cultures requiring further evaluation and intravenous antibiotics. The time to intentional admission was 2.3 days in the pre-implementation group and 0.8 days in the post-implementation group.

## 4. Discussion

This study demonstrated a 36.3% reduction in time to result review with a pharmacist-driven program for culture follow-up in the ED when compared to the administrative staff-physician collaborative driven model. This impact can be attributed both to the live-alert workflow, the confidence and knowledge of RDTs and interpretation of results performed by a pharmacist to make decisions and recommendations to the ED provider based on preliminary culture results. This reduction in time to result review led to a reduction in time to first patient contact attempt and time to intervention. Although the 19.2% reduction in time to intervention was not statistically significant, there may be clinical significance of this 21-h reduction in time to intervention. Of the 22 patients who experienced a repeat ED encounter, 11 occurred within seven days of the index encounter discharge. For these patients, time to repeat ED encounter ranged from 13.1 to 163.2 h. A 21-h reduction in time to intervention could be impactful in reducing ED visits.

Positive STI results comprised 31.3% of all positive results reviewed during the study period, and 30.7% of all positive results requiring outpatient intervention. This finding highlights a potential missed opportunity for empiric STI treatment(s) during the initial ED encounter in select high-risk patients per the Centers for Disease Control and Prevention (CDC) guideline recommendations [15]. The practice of withholding treatment for STIs until results are obtained often leads to limited treatment options (e.g., limited to oral prescriptions) or inability to connect with the patient for follow-up, especially among patients seeking primary care in the ED. Additionally, the post-implementation group had 14.7% more outpatient follow-ups due to a PCR/molecular testing result. As this is the method for STI testing that was consistently used across the time of the study as it became the standard testing modality for STIs prior to this study, this may indicate an increased rate of withholding empiric STI treatment.

A significantly higher proportion of patients in the post-implementation group were not able to be reached after several telephone attempts (at a minimum, two telephone calls were attempted), and therefore required a letter to be sent to the patient’s residence for an attempt at outpatient follow-up (4.7% vs. 19%, p = 0.026). Other published studies suggest that the rate of unsuccessful patient contact via telephone ranges from 7% to 57% [16]. There are certain confounders that could have contributed to the difference demonstrated in this study, including the daily time frame during which patient contact is attempted and the lack of a consistent workspace and telephone. The lack of a designated phone and the inconsistency of the pharmacy resident’s workspace could have potentially contributed to the decreased success in patient contact by telephone. The allocation of a designated pharmacist for this process and development of this pilot into a permanent workflow could address this issue.

There were no significant differences noted in outcomes related to repeat ED encounters and hospital admissions within 30 days between the two groups (Table 3). Due to the constraints of the study period, the comparative numbers were relatively low, with a total of 22 patients experiencing a repeat ED encounter and 11 patients experiencing a hospital admission. When evaluating the reasons for repeat hospital encounters and hospital admissions, there were fewer patients in the post-implementation group who had subsequent events that were related to the index case (similar chief complaint). Although it is difficult to draw conclusions from this small subgroup of patients, these results may signal a longer study period and more robust comparisons may show that pharmacist-performed follow-up helps prevent repeat ED encounters and hospital admissions related to the index case.

The pharmacist-driven approach also demonstrated a significant reduction in prescribing of fluoroquinolones during outpatient follow-up. Fluoroquinolone prescribing occurred exclusively for the patient population with positive urine cultures. Per Infectious Disease Society of America’s (IDSA) guidelines and the local antimicrobial prescribing guidelines, fluoroquinolones should be reserved for patients with signs and symptoms of pyelonephritis, such as fever >38 °C and unilateral back or flank pain. This reduction in fluoroquinolone prescribing can be explained by the addition of a thorough chart review and recommendation by a pharmacy resident with stewardship training and experience. Prior to the introduction of a pharmacist-driven, multidisciplinary approach, a physician was the sole clinician responsible for chart review and the outpatient follow-up plan for identified cultures across the three hospital system, potentially contributing to over prescribing of fluoroquinolones (such as for acute cystitis and asymptomatic bacteriuria). This pilot led the implementation of active, formal ASP activity in the ED. Prior to this pilot, ASP activities centered around education on local prescribing guidelines and resistance scores [17,18]. While providers, predominantly residents, are educated on these resources, they are not required to use them, which may limit their effectiveness. Active and patient-specific incorporation of these resources through ASP activities such as pharmacist-driven culture follow-up will be needed to impact change in antibiotic utilization. Furthermore, the implementation of this pilot relied on a pharmacy resident to carry out daily review and interventions. The results of this study highlight the capacity of a pharmacy trainee to conduct ASP activities and support the reframing of resources for longitudinal ASP activities in the ED, a possible new avenue for a pharmacy resident’s clinical experiences.

Limitations of this study include the lack of resources available for execution of the pilot, the small sample size, that pharmacist time spent during the pilot was not studied, and that power criteria were not met. The sample size could have been expanded to include cultures across the three campus systems and a longer time interval to conduct the pilot if there was additional pharmacist support and information technology support allocated to the pilot. Unfortunately, pharmacist time spent during the pilot was not examined, limiting the ability to determine an appropriate proportion of this responsibility to designate to a new or existing pharmacy service. It was difficult to accurately measure time spent during the pilot due to the segmented approach to patient care due to other daily patient care responsibilities, which were triaged daily by the pharmacy resident. The pharmacy resident estimates approximately six additional hours of work per week designated to the pilot.

## 5. Conclusions

Introduction of a pharmacist-driven culture and STI test follow-up program in the ED at a tertiary teaching hospital executed by a pharmacy resident demonstrated a reduction in time to result review and intervention. Additionally, fluoroquinolone prescribing was significantly reduced for patients with positive urine cultures. Full implementation of a pharmacy service could optimize stewardship initiatives and improve antimicrobial selection through the addition of a pharmacist trained in stewardship.

## Figures and Tables

**Table 1:** Baseline Characteristics.

Baseline Characteristic, n (%)	Pre-Implementation, n = 64	Post-Implementation, n = 63	p-Value
Mean age, years (standard deviation)	50.67 (23.4)	42.40 (24.0)	0.028
Female gender	51 (79.69)	51 (80.95)	1
**Result type**			0.17
Urine	46 (71.9)	36 (57.1)	
PCR/molecular testing ^1^	15 (23.4)	24 (38.1)	
Blood cultures	3 (4.7)	3 (4.8)	
**Antibiotic provided upon ED discharge**			0.35 ^2^
Nitrofurantoin	4 (6.3)	7 (11.1)	
Sulfamethoxazole/trimethoprim	9 (14.1)	3 (4.8)	
Clindamycin	0	1 (1.6)	
Metronidazole	9 (14.1)	6 (9.5)	
No antibiotic	38 (59.4)	38 (60.3)	
Other	1 (1.6)	0	
Beta-lactam	1 (1.6)	2 (3.2)	0.62
Fluoroquinolone	2 (3.1)	4 (6.4)	0.44

^1^ RDT for *N. gonorrhoeae*, *C. trachomatis*, and *T. vaginalis*. ^2^ p-value evaluating for differences in the following antimicrobials as a group: nitrofurantoin, sulfamethoxazole, clindamycin, metronidazole, other antimicrobial, and no antimicrobial prescribed. Beta-lactam and fluoroquinolones were excluded from this specific analysis and p-values comparing these groups of antimicrobials are listed.

**Table 2:** Types of outpatient interventions.

Type of Intervention, n (%)	Pre-Implementation, n = 64	Post-Implementation, n = 63	p-Value
Initiation of therapy	25 (39)	19 (30.2)	0.39
Modification of therapy	24 (37.5)	14 (22.2)	0.09
Contact primary care provider	4 (6.3)	8 (12.7)	0.35
Contact nursing home	5 (7.8)	7 (11.1)	0.74
No medication ^1^	5 (7.8)	5 (7.9)	1
Letter sent	3 (4.7)	12 (19)	0.026

^1^ Interventions that resulted in contact with patient with no change or initiation of new therapy.

**Table 3:** Repeat ED Encounters and Hospital Admissions.

Outcome, n (%)	Pre-implementation, n = 64	Post-implementation, n = 63	p-Value
Patients with repeat ED encounter within 30 days	10 (15.6)	12 (19.1)	0.783
Patients with repeat ED encounter related to index case	5 (7.3)	0	--
Patients with hospital admissions within 30 days	6 (9.4)	5 (7.9)	1
Patients with unintended repeat hospital admission related to index case	2 (2.9)	1 (1.7)	--

**Figure A1:**
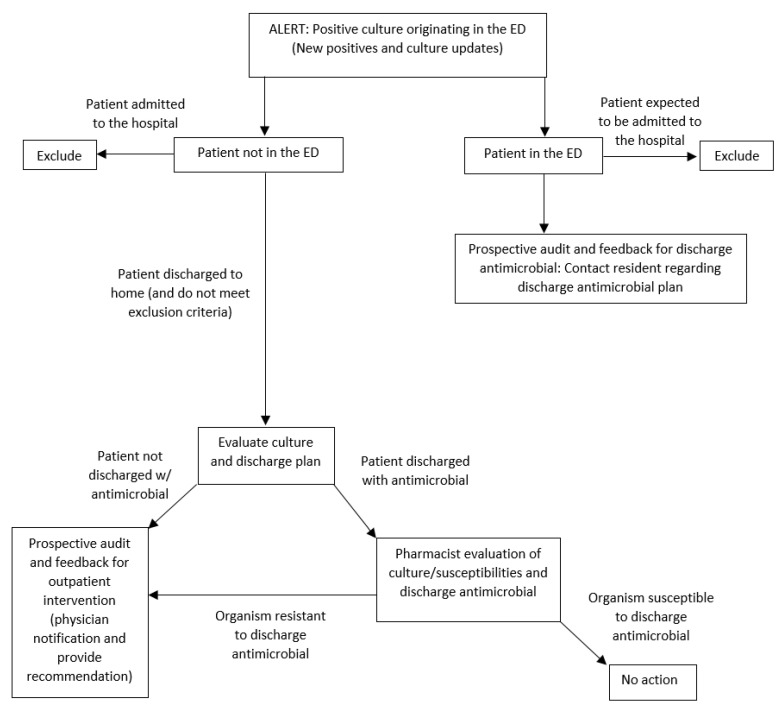
Review and Recommendation Algorithm.
